# Evaluation of the Apnea-Hypopnea Index Determined by Adaptive Servo-Ventilation Devices in Patients With Heart Failure and Sleep-Disordered Breathing

**DOI:** 10.3389/fcvm.2021.680053

**Published:** 2021-06-25

**Authors:** Satomi Imanari, Yasuhiro Tomita, Satoshi Kasagi, Fusae Kawana, Yuka Kimura, Sugao Ishiwata, Koji Narui, Takatoshi Kasai

**Affiliations:** ^1^Sleep Center, Toranomon Hospital, Tokyo, Japan; ^2^Clinical Physiology, Toranomon Hospital, Tokyo, Japan; ^3^Cardiovascular Center, Toranomon Hospital, Tokyo, Japan; ^4^Cardiovascular Respiratory Sleep Medicine, Juntendo University Graduate School of Medicine, Tokyo, Japan; ^5^Department of Cardiovascular Medicine, Juntendo University Graduate School of Medicine, Tokyo, Japan; ^6^Okinaka Memorial Institute for Medical Research, Tokyo, Japan

**Keywords:** apnea, hypopnea, polysomnography, titration, heart failure, adaptive servo-ventilation

## Abstract

**Introduction:** Adaptive servo-ventilation (ASV) devices are designed to suppress central respiratory events, and therefore effective for sleep-disordered breathing (SDB) in patients with heart failure (HF) and provide information about their residual respiratory events. However, whether the apnea-hypopnea index (AHI), determined by the ASV device AutoSet CS (ASC), correlates with the AHI calculated by polysomnography (PSG) in patients with HF and SDB remains to be evaluated.

**Methods:** Consecutive patients with SDB titrated on ASC were included in the study. We assessed the correlation between AHI determined by manual scoring during PSG (AHI-PSG) and that determined by the ASC device (AHI-ASC) during an overnight session.

**Results:** Thirty patients with HF and SDB (age, 68.8 ± 15.4 years; two women; left ventricular ejection fraction, 53.8 ± 17.9%) were included. The median AHI in the diagnostic study was 28.4 events/h, including both obstructive and central respiratory events. During the titration, ASC markedly suppressed the respiratory events (AHI-PSG, 3.3 events/h), while the median AHI-ASC was 12.8 events/h. We identified a modest correlation between AHI-PSG and AHI-ASC (*r* = 0.36, *p* = 0.048). The Brand-Altman plot indicated that the ASC device overestimated the AHI, and a moderate agreement was observed with PSG.

**Conclusions:** There was only a modest correlation between AHI-PSG and AHI-ASC. The discrepancy may be explained by either the central respiratory events that occur during wakefulness or the other differences between PSG and ASC in the detected respiratory events. Therefore, clinicians should consider this divergence when assessing residual respiratory events using ASC.

## Introduction

Sleep-disordered breathing (SDB) is common in patients with heart failure (HF) and sometimes consists of obstructive and central events (1). Continuous positive airway pressure (CPAP) devices are available for treating SDB, but are less effective for central sleep apnea (CSA) than for obstructive sleep apnea (OSA). Approximately half of patients with CSA have residual respiratory events [apnea-hypopnea index (AHI) ≥ 15 events/h] (2). Some patients with OSA on CPAP devices develop CSA, previously reported as complex sleep apnea (3), which is now called as treatment-emerged CSA and often observed in patients with HF.

Adaptive servo-ventilation (ASV) is an effective treatment for treatment-emerged CSA and thus can be useful in treating SDB in patients with HF (4). Furthermore, ASV devices provide information about the residual respiratory events. However, there are no data regarding the accuracy of AHI determined by the ASV device AutoSet CS™ (ASC; ResMed Ltd., Bella Vista NSW, Australia) compared with AHI calculated by polysomnography (PSG) in patients with HF and SDB. A previous study (5) demonstrated a strong correlation between AHI determined by manual scoring and that calculated by CPAP devices; however, patients with HF were excluded from this study.

Based on the above, this study aimed to compare AHI determined by the ASC device (AHI-ASC) with that calculated by PSG (AHI-PSG) to evaluate the performance of ASC. The study results will help us identify residual respiratory events in patients with HF and SDB using ASV devices.

## Methods

### Patients

We have defined patients with heart failure as those with the diagnosis of heart failure based on attending cardiologists and those with a history of hospitalization for decompensated heart failure. Patients with symptomatic but stable HF who underwent titration PSG using the ASV device AutoSet CS™ (ASC; ResMed Ltd., Bella Vista NSW, Australia) at our sleep center (Toranomon Hospital, Tokyo, Japan) between March 2009 and February 2013 were enrolled. All patients had moderate-to-severe SDB, defined as AHI ≥ 15 events/h of sleep on diagnostic PSG before titration PSG. The ethics committee of Toranomon Hospital approved this study (No. 1833).

### Measurements

All PSGs were performed by a sleep technician using a digital polygraph (SomnoStar α Sleep System; SensorMedics Corp., Yorba Linda, CA, USA). Before titration of PSG using ASC, a diagnostic study was performed for each patient. Accepted definitions and scoring methods for the diagnosis of SDB have been used for both diagnostic PSG and titration PSG (6). In particular, apnea was defined as a > 90% decrease in airflow for at least 10 s, and hypopnea was defined as a 30% decrease in airflow with an associated 3% oxygen desaturation for at least 10 s.

During the second PSG, air flow signals were obtained from the nasal mask of the ASC device. Titration procedures were as follows: first, the end-expiratory pressure level was titrated manually to alleviate upper airway obstruction and, second, the minimum and maximum pressure support levels were also titrated if there were central respiratory events. Respiratory events were manually scored for each titration PSG, and AHI (AHI-PSG) was calculated per hour of sleep according to the definition used in the diagnostic PSG. AHI determined by the ASC device (AHI-ASC) was calculated as the total number of apnea and hypopnea per hour of the period on the ASC. The ASC device automatically detected and recorded respiratory events according to the following definitions: apnea events were scored when there was a >75% decrease in the recent average (time constant of 2 s) of the tidal volume for >10 s compared with the baseline (moving average of 100 s prior to the period of recent average) and hypopnea events were scored when there was a >50% decrease in the recent average of the tidal volume for >10 s compared with the baseline. We downloaded the AHI-ASC using the ResLink device after each PSG titration. In addition, the ASC device reported overnight summary data for leakage (maximum, 95th percentile, and median level of leakage) and pressure levels (expiratory and inspiratory pressure).

Moreover, we conducted two additional modified AHI-PSG scoring systems. Since patients with HF (especially those predominantly presenting CSA) frequently have respiratory events while awake, the difference between total sleep time (TST) during PSG and the recording period on ASC may result in a discrepancy between AHI-PSG and AHI-ASC. Therefore, we repeated the counting of all respiratory events during the period on ASC [i.e., recording time while on ASC (RT)], including the awakening periods, and divided the number of these events by the RT (AHI-PSG-RT). Moreover, because the ASC device detects events based only on the flow signal, we again scored all respiratory events based only on flow signals during RT, thus ignoring the discernible reduction of airflow with oxygen desaturation or arousal. The number of events was divided by the RT (AHI-PSG-F).

### Statistical Analysis

Data are expressed as mean ± standard deviation or median (interquartile range) for continuous variables and numbers and percentages for categorical variables. The data obtained by PSG were compared with those retrieved from the ASC device using the Wilcoxon rank-sum test, correlation analysis, and Bland and Altman plots (7). The same analyses were performed to compare the modified AHI (AHI-PSG-RT and AHI-PSG-F) and AHI-ASC. A *p*-value < 0.05 was considered as the level of statistical significance.

## Results

Overall, 30 patients were included in this study. The patient characteristics are shown in Table 1. The mean age was 68.8 years, and 93% of the patients were men. The mean body mass index (BMI) was 24.0. The mean left ventricular ejection fraction was 53.8%, indicating that the study included patients with a preserved ejection fraction. Fifty-seven percent of the patients had a history of persistent or permanent atrial fibrillation. The etiology of HF was ischemic in 43% of patients.

**Table 1:** Patient characteristics.

Age, years	68.8 ± 15.4
Men, *n* (%)	28 (93)
BMI, kg/m^2^	24.0 ± 4.9
LVEF, %	53.8 ± 17.9
HFrEF, *n*%	8 (27)
BNP, pg/mL	220 (489)
Creatinine, mg/dL	1.1 (0.5)
**Complications**	
AF, *n* (%)	17 (57)
Ischemic etiology, *n* (%)	13 (43)
**Medications**	
Beta blockers, *n* (%)	14 (47)
RAS inhibitors, *n* (%)	14 (47)
Diuretics, *n* (%)	12 (40)

*AF, atrial fibrillation; BMI, body mass index; BNP, brain natriuretic peptide; HFrEF, heart failure with reduced LVEF; LVEF, left ventricular ejection fraction; RAS, renin-angiotensin system.*

*RAS inhibitors included angiotensin converting enzyme inhibitors, angiotensin receptor blockers, and aldosterone blockers.*

*Reduced LVEF was defined as LVEF <40%*.

Diagnostic PSG findings are shown in Table 2. The mean TST during PSG was 300 ± 88 min. The median AHI was 28.4, and 50% of the patients had a dominant central SDB (central AHI was higher than obstructive AHI). The findings from the titration PSG and the ASC device are summarized in Table 3.

**Table 2:** Findings of the diagnostic polysomnography.

TST, min	300 ± 88
AHI, events/h of sleep	28.4 (29.1)
OAHI, events/h of sleep	7.0 (13.5)
MAHI, events/h of sleep	0.1 (5.4)
CAHI, events/h of sleep	12.4 (27.6)
AI, events/h of sleep	25.7 (38.9)
HI, events/h of sleep	8.9 (13.6)
Arousal index, events/h of sleep	39.7(29.7)
Lowest SpO_2_, %	82.4 ± 6.7
**Sleep stage, % of TST**	
SWS	1.3 (6.9)
REM	9.7 (6.7)

*AHI, apnea-hypopnea index; TST, total sleep time; CAHI, central AHI; HI, hypopnea index; MAHI, mixed AHI; OAHI, obstructive AHI; AI, apnea index; SWS, slow-wave sleep; REM, rapid eye movement*.

**Table 3:** Data from the ASC, polysomnographic findings, and additional modified scorings of respiratory events during ASV titration.

**ASC report**	
RT, min	448 ± 53
AHI-ASC, events/h of sleep	12.8 (11.8)
AI-ASC, events/h of sleep	2.8 (6.6)
HI-ASC, events/h of sleep	8.3 (7.3)
**Pressure, cmH**_**2**_**O (95th percentile)**	
Mean pressure	8.2 ± 2.3
End-expiratory pressure	7.4 ± 2.1
Peak inspiratory pressure	9.3 ± 6.9
**Leak, L/min**	
Maximum	118 (25)
95th percentile	16 (30)
Median	0 (6.0)
**PSG findings**	
TST, min	300 ± 88
AHI-PSG, events/h of sleep	3.3 (5.3)
AI-PSG, events/h of sleep	0.0 (0.9)
HI-PSG, events/h of sleep	3.0 (4.1)
**Modified PSG findings**	
AHI-PSG-RT	6.4 (5.7)
AHI-PSG-F	8.8 (9.3)

*RT, recording time; ASC, AutoSet CS; AHI-ASC, apnea-hypopnea index determined by the ASC device; AI-ASC, apnea index determined by ASC device; HI-ASC, hypopnea index determined by the ASC device; TST, total sleep time; AHI-PSG, apnea-hypopnea index assessed by polysomnography; AI-PSG, apnea index assessed by polysomnography; HI-PSG, hypopnea index assessed by polysomnography; AHI-PSG-RT, modified apnea-hypopnea index determined by polysomnography after recounting the number of all respiratory events during the recording time; AHI-PSG-F, modified apnea-hypopnea index determined by polysomnography after rescoring all respiratory events only based on flow signal during the recording time*.

**Figure 1:**
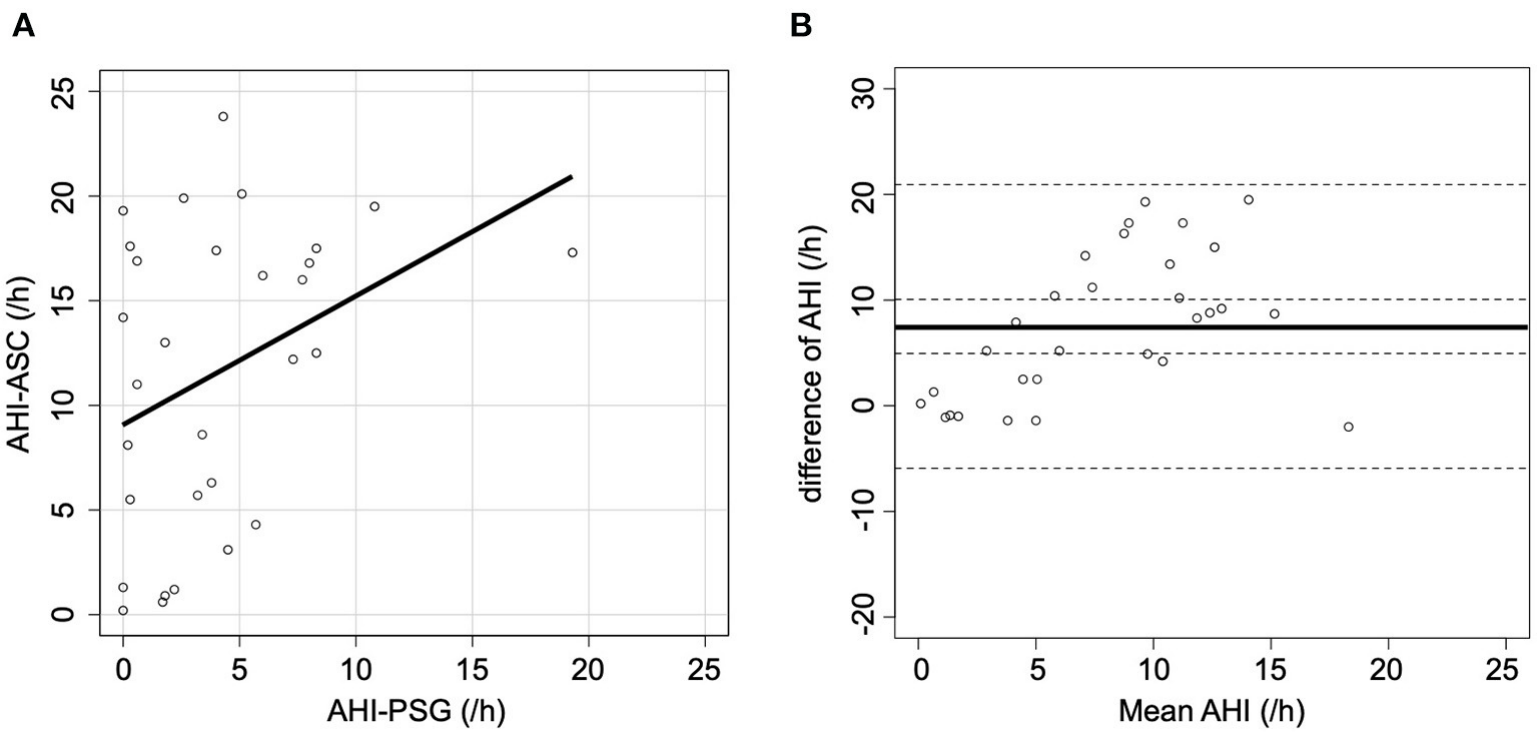
Scatter and Bland-Altman plots comparing AHI-PSG and AHI-ASC. **(A)** There was a modest correlation between AHI-PSG and AHI-ASC. **(B)** The Y-axis indicates the difference between AHI-PSG and AHI-ASC [(AHI-PSG)–(AHI-ASC)]. The X-axis indicates the mean values of AHI. The solid line represents the mean difference, while the dashed lines represent the limit of agreement (± 2 SD).

Figure 1A shows the correlation between AHI-PSG and AHI-ASC, which was modest (*r* = 0.36; *p* = 0.048). Figure 1B illustrates a Bland-Altman plot that shows a mean AHI difference of 7.5 events/h of sleep between the two methods; the limits of agreement for AHI ranged between −5.9 and 20.9.

**Figure 2:**
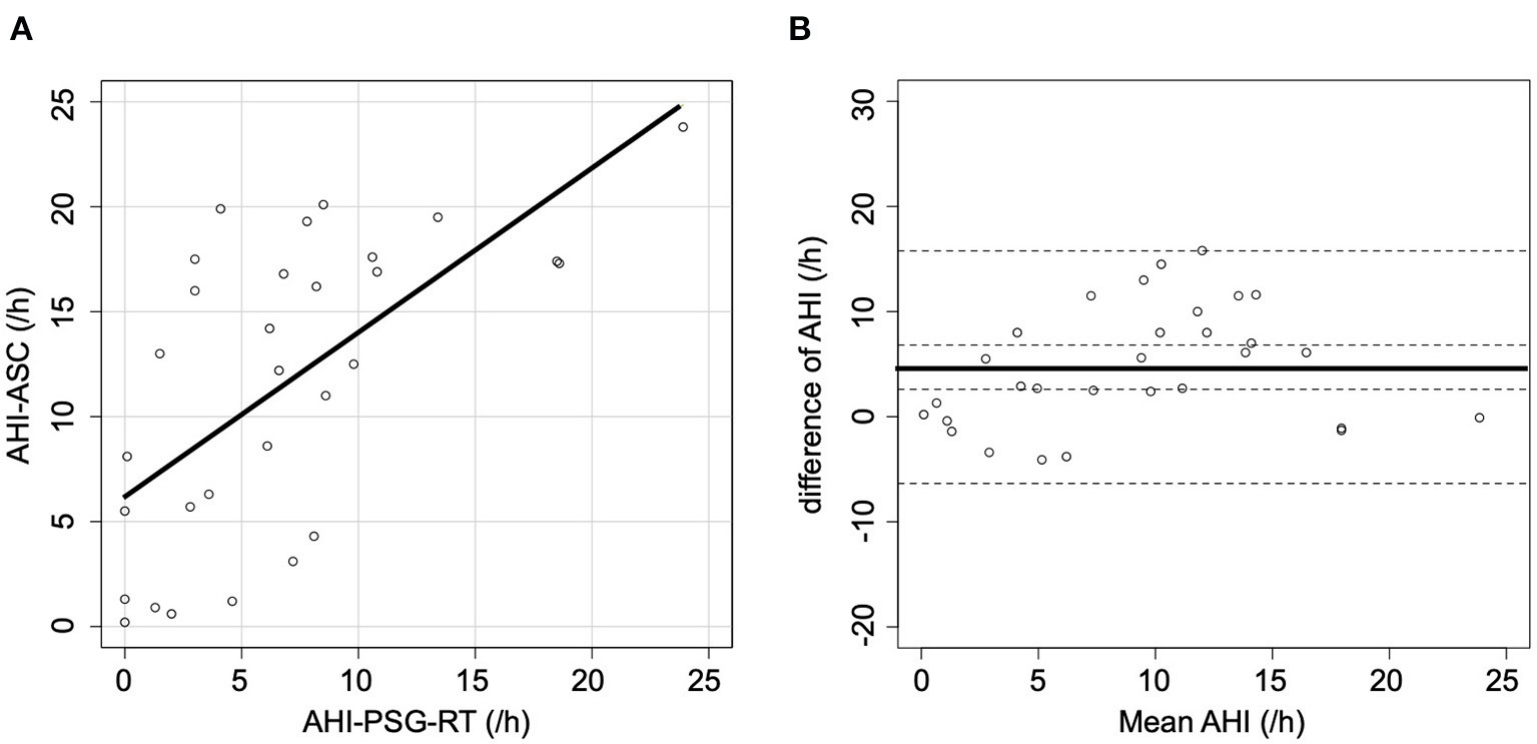
Scatter and Bland-Altman plots comparing AHI-PSG-RT and AHI-ASC. **(A)** There was a moderate correlation between AHI-PSG-RT and AHI-ASC. **(B)** The Y-axis represents the difference between AHI-PSG-RT and AHI-ASC [(AHI-PSG-RT)–(AHI-ASC)]. The X-axis indicates the mean values. The solid line represents the mean difference, while the dashed lines represent the limit of agreement (± 2 SD).

**Figure 3:**
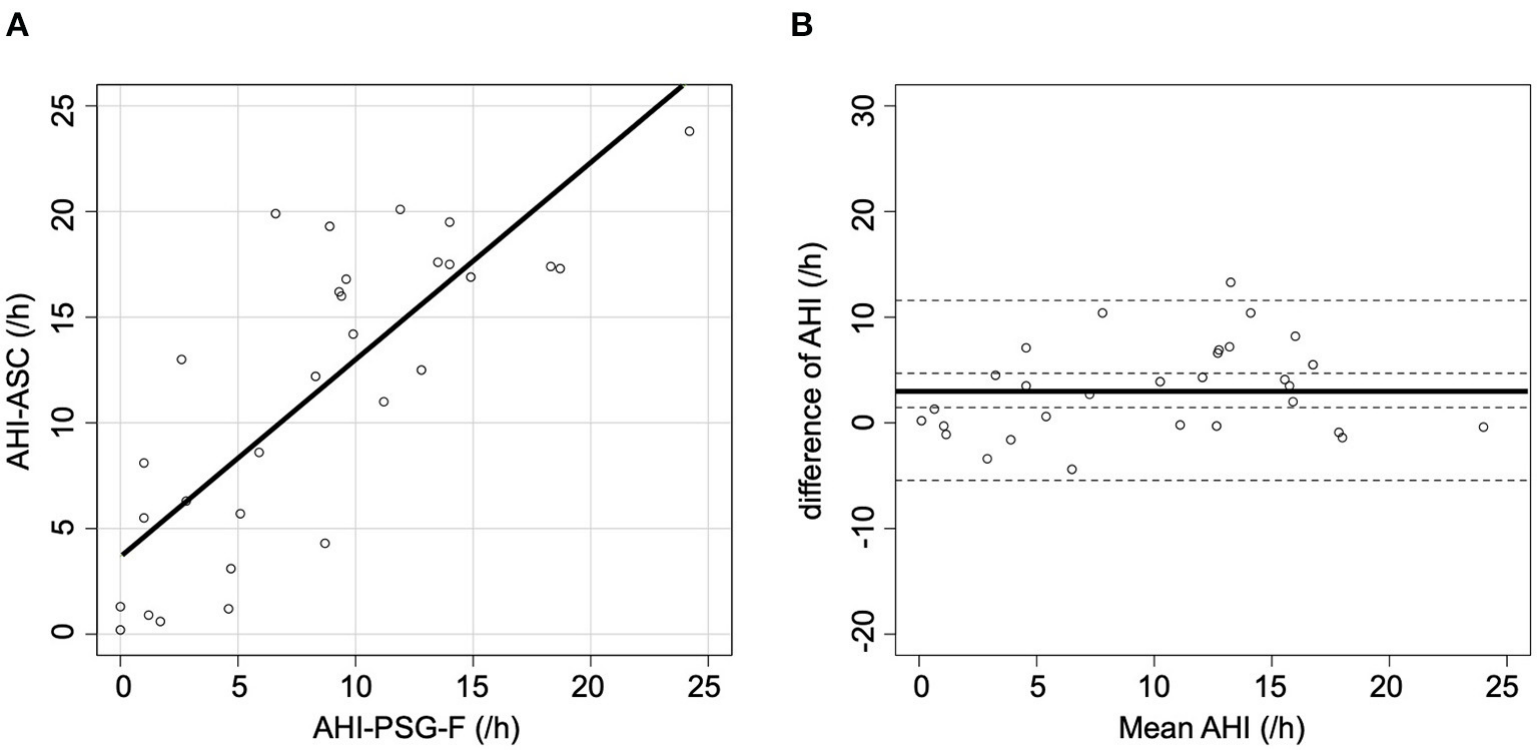
Scatter and Bland-Altman plots comparing AHI-PSG-F and AHI-ASC. **(A)** There was a moderately strong correlation between AHI-PSG-F and AHI-ASC. **(B)** The Y-axis indicates the difference between AHI-PSG-F and AHI-ASC [(AHI-PSG-F)–(AHI-ASC)]. The X-axis indicates the mean values. The solid line represents the mean difference, while the dashed lines represent the limit of agreement (± 2 SD).

When AHI-PSG was recomputed, including the periods of awakening, the correlation with AHI-ASC improved (*r* = 0.64; *p* < 0.001, Figure 2A). Figure 2B presents a Bland-Altman plot that shows a mean AHI difference of 4.7; the limits of agreement for AHI ranged from −6.4 to 15.8. Furthermore, when all respiratory events in PSG were rescored based only on the flow signal, the correlation with AHI-ASC improved (*r* = 0.80; *p* < 0.001, Figure 3A). Figure 3B shows a Bland-Altman plot in which the mean AHI difference was 3.1; the limits of agreement for AHI ranged from −5.4 to 11.6.

## Discussion

Two important observations were made in this study. First, there was a modest but significant correlation between AHI-PSG and AHI-ASC, with a mean difference of 7.5 events/h. This suggest that the residual AHI provided by the ASC device (i.e., AHI-ASC) overestimated the actual residual AHI (i.e., the AHI-PSG) by 7.5 events/h. Second, there was a proportional error between the AHI-PSG and the corrected AHI-ASC, but the agreement was improved, and the proportional error was also improved after recounting the number of all respiratory events during the recording time. Further improvement was observed in the agreement after rescoring all respiratory events only based on flow signal during the recording time. These findings provide a possible explanation for the modest correlation and agreement between AHI-ASC and the original AHI-PSG. Notably, manual scoring was performed for TST in PSG, whereas the report from ASC was based on RT, including latency to sleep onset and nocturnal awaking time. Patients with HF sleep less on average than those in the general population (8). Consequently, AHI-PSG would be expected to be underestimated by the ASC device because the denominator of AHI-ASC was RT, which was longer than the TST. However, AHI-ASC was overestimated compared to the original AHI-PSG. Based on these findings, we added a scoring of AHI-PSG based on the RT (i.e., AHI-PSG-RT) and found a better correlation and smaller difference between AHI-ASC and AHI-PSG-RT. In addition, patients with HF are likely to have a periodic breathing pattern not only while sleeping, but also while awake (9). Silva et al. (10) demonstrated that periodic breathing while awake before sleep onset was detected in 44% of patients with HF by PSG. The presence of periodic breathing while awake may play a role in the discrepancy between AHI-PSG and AHI-ASC. The ASC device only detects flow signal attenuation and hence is likely to overestimate the number of hypopnea episodes compared with PSG, in which hypopnea is only scored when flow attenuation is accompanied by oxygen desaturation or arousal. SDB in patients with HF includes central respiratory events, which have been reported to be associated with decreased oxygen desaturation (11, 12). Moreover, patients with HF are likely to have flow attenuation even when awake. Therefore, flow attenuations even without oxygen desaturation or arousal would be scored as hypopnea by ASC, but would not be scored in PSG. Furthermore, the definitions of flow attenuation were different between the two approaches: flow attenuation in PSG was defined as a decrease in the amplitude of the flow signal, while flow attenuation in the ASC was defined as a decrease in the tidal volume. The baseline for the tidal volume in the case of ASC and the amplitude of the flow signal in the case of PSG were defined as 100 and 120 s, respectively. These differences may also affect the sensitivity of detecting the flow attenuation.

Although PSG remains the gold standard, respiratory events detected by ASCs are also important. AHI-ASC may include respiratory events in the periodic breathing pattern while the patient is awake because the device is incapable of recognizing sleep. Since this breathing pattern during wakefulness is associated with a poor prognosis in patients with HF (13, 14), AHI-ASC may have a prognostic value similar to that determined for patients on CPAP in the Central Sleep Apnea and Heart Failure Trial (CANPAP study) (2). Further studies are required to investigate the prognostic impact of residual AHI as determined by ASC.

Currently, newer models of ASV are available, but some people continue to use ASCs. Therefore, we believe that the results of this study will have an impact on the interpretation of residual AHI. In addition, considering the importance of periodic breathing patterns while awake, we can apply this concept to newer ASV devices.

A recent randomized control trial (15) has suggested that ASV may be harmful in heart failure with low cardiac function with central sleep apnea. They have speculated that poor adherence to ASV and the possibility that the high set pressure has reduced cardiac output may have negatively affected. We speculated from our study that titrating the ASV based on the AHI calculated from only flow alternations may lead to unnecessary pressure elevation related to decreased adherence for ASV and decreased stroke volume. Heart failure with reduced ejection fraction is often associated with CSA, and therefore, future research will be needed to verify the safety of ASV for heart failure patients with CSA. ASV is also expected to be effective in other situations where CSA may occur, such as in the acute phase of myocardial infarction (16).

This study had several limitations. Because the residual AHI, AHI-ASC, was not very high in the present study, our results should be interpreted with caution, especially in patients with many residual events, because different mechanisms may be implicated. Moreover, we only compared total AHI and did not distinguish between central and obstructive AHI because at the time of the study, ResScan did not provide central and obstructive AHI separately. Therefore, similar investigations using the newest version of ResScan software and ASC devices are necessary. Furthermore, since most of the participants in this study were male, we should refrain from applying our conclusions to female patients.

In conclusion, there was only a modest correlation between the AHI-PSG and AHI-ASC. This discrepancy may be explained by the central respiratory events during wakefulness or by the attenuation of flow signal amplitude without either arousal or oxygen desaturation, both of which are often observed in patients with HF. Therefore, clinicians should consider this discrepancy when assessing residual AHI using ASC devices.

## Data Availability Statement

The raw data supporting the conclusions of this article will be made available by the authors, without undue reservation.

## Ethics Statement

The studies involving human participants were reviewed and approved by the ethics committee of Toranomon Hospital. Written informed consent for participation was not required for this study in accordance with the national legislation and the institutional requirements.

## Author Contributions

SIm was responsible for the rescoring polysomnography and data management, as well as contributed to the manuscript writing. YT is the guarantor of the paper and takes responsibility for the integrity of the work as a whole. YK was responsible for data management and manuscript writing. FK was responsible for the rescoring polysomnography and contributed to the manuscript writing. SK was responsible for data management and reviewing and editing the manuscript. SIs and KN was responsible for data management and reviewing and editing the manuscript. TK was responsible for the research design, data analysis and interpretation, and manuscript writing. All authors contributed to the article and approved the submitted version.

## Conflict of Interest

TK is affiliated with a department endowed by Philips Respironics, ResMed, Teijin Home Healthcare, and Fukuda Denshi. The remaining authors declare that the research was conducted in the absence of any commercial or financial relationships that could be construed as a potential conflict of interest.
